# Pre-contact by telephone increases response rates to postal questionnaires in a population of stroke patients: an open ended randomized controlled trial

**DOI:** 10.1186/s12913-016-1732-8

**Published:** 2016-09-21

**Authors:** Mathias Barra, Tone Breines Simonsen, Fredrik Andreas Dahl

**Affiliations:** Akershus University Hospital, Health Services Research Center, Sykehusveien 25, Lørenskog, 1473 Norway

**Keywords:** Response rates, Postal survey, Questionnaires, Randomized controlled trial, Pre-contact, Stroke patients

## Abstract

**Background:**

A follow-up study on a cohort of stroke patients through a postal survey questionnaire 3 and 12 months after discharge from hospital was performed. The response rate at 3-months follow-up was lower than desired, and pre-contact by phone as a measure for increasing the response rate at 12 months was studied.

**Methods:**

The study design was a randomized controlled trial on a cohort of 3 months follow-up-non-responders where the intervention group was pre-contacted with an aim to obtain an informal ’consent to receive’ the questionnaire before the 12-months survey was mailed, and the control group was not.

The primary outcome was 45 days response rate; secondary outcome was 365 days response rate. The main analysis followed the intention to treat principle. A secondary, per-protocol analysis (i.e. subjects who were not reached by phone were reassigned to the control group) is included. Also included is a rudimentary cost-utility analysis, where we estimated the cost per additional response.

**Results:**

Of the 235 subjects, 116 were randomized to the intervention group and 119 to the control group. 10 were excluded due to death (7 in the IG and 3 in the CG), 6 due to dementia (3 in the IG and 3 in the CG), and 2 (1 in the IG and 1 in the CG) for other reasons. The primary outcome was a response rate of 42.9 % in the intervention group, and 26.8 % in the control group, giving *p* =0.014, with estimated OR of 2.04 (95 % CI [1.16,3.64]). The secondary outcome had *p* =0.009 with OR 2.10 (95 % CI [1.20,3.70]). The as-per-protocol analyses gave stronger results with *p* =0.001 and *p* =0.003, respectively. The cost-utility analysis gave a time cost of 1 working hour per additional response.

**Conclusions:**

The results are in line with previous research, and show that pre-contact has a positive effect on response rate also for a population of elderly with reduced health. Given the importance of high response rate in surveys, a cost of 1 working hour per additional response is likely to be worth while.

**Trial registration:**

Registration with ISRCTN initiated on 05/21/2013 and finalised on 06/30/2014 with http://www.isrctn.com/ISRCTN31304930. Following the prospective submission in May 2013, there were no subsequent changes to the protocol. The recruitment started on 01/06/13, after initiation of public registration.

## Background

### Collecting patient data

Certain kind of patient data relevant to health care researchers can only be harvested from the patients themselves, e.g. *health related quality of life* or *treatment satisfaction*. While non-subjective patient data, e.g. *number of visits with a GP* or *hours of home care per week received*, may be possible to obtain from other sources in theory, postal or electronic survey questionnaires often stand out as the cost-effective alternative ([1], p. 2). Once a survey questionnaire design is chosen, researchers should – subject to their available resources – undertake to maximize the *response rate (RR)*. Low RR’s obviously decrease the statistical power of the final data set, increasing type II errors. Worse yet; if propensity for (non-)responding correlates with any of the collected variables, subsequent analysis will usually be biased, which increases the risk of Type I errors. This problem is particularly acute if an outcome variable is causally linked to response propensity. There is substantial evidence that responders and non-responders differs with respect to a range of characteristics; see e.g. [2] for an introduction and further references.

### **NORSPOT** – the context of the RCT

NORSPOT (*Norwegian Stroke – Paths of Treatment*) is an established cohort consisting of all patients admitted to the Stroke Unit (SU) of Akerhus University Hospital (Ahus) during the period February 15th 2012 to March 15th 2013. Ahus is situated in the Oslo greater metropolitan area, and is Norway’s largest acute hospital with a catchment area counting circa 500.000 inhabitants (≃10 % of Norway’s population). The project’s charter is to map out stroke patients’ treatment paths through the health services, and to estimate current epidemiological data, with an aim to inform policy makers about future needs within the various parts of the Norwegian health care system. To this end, in addition to data collected during hospitalization, NORSPOT seeks to elicit information about the patients’ levels of, contentment with, and benefit from, consumption of health services over the year following hospitalization. Postal survey questionnaires were therefore sent to the patients’ home address at 3 and 12 months after the time of discharge from the SU. The data set contains 1144 unique patients with a stroke diagnosis. The project recorded data on circa 1900 admissions to the SU. There were several patients with multiple stays, and about two thirds of all patients admitted to the SU received a final stroke diagnosis. Here, the stroke diagnoses are classified into intra-cranial haemorrhages (ICD10 61.X), cerebral infarctions (ICD10 63.X) and transient ischemic attacks (ICD10 45.X excluding 45.4). Patient’s with other diagnoses were not included in the follow-up questionnaire study. During the 3 month survey questionnaire phase, we observed that the response rates were converging towards the lower end of the acceptable range (≃50 %), and we were worried that further *loss-to-follow-up* during the 12 month questionnaire phase could weaken our study.

We therefore searched the literature on increasing response rates to postal questionnaires to see if there were any easy and efficient steps which could be implemented to maintain – or even improve – the state of affairs.

### Review of literature on increasing response rates by pre-contact

A Cochrane review from 2010 [1] identified and analysed 481 papers investigating different strategies to increase response rates to postal questionnaire surveys. We noted that amongst the diverse approaches shown to have a positive effect on response rates, many were already implemented (e.g. handwritten address, university hospital envelope), while other approaches were either considered unethical according to local ethical considerations (pecuniary incentives) or incompatible with our study (short questionnaire, non-sensitive questions).

One intervention singled itself out: *pre-contact*. Pre-contact here means that the prospective respondents are contacted prior to having the questionnaire mailed to them. The Cochrane report reviewed various studies: pre-contact by mail (e.g. a postcard one week prior to the actual questionnaire) or pre-contact by telephone (i.e. calling the respondents prior to sending them the questionnaire). In addition, some comparisons of these two forms of pre-contact was reported on.

The strategy of pre-contact by telephone was both feasible to implement – at least partially – on NORSPOT’s budget, and ethical approval for the intervention had been obtained already; indeed pre-contact was already implemented for the most frail patients (e.g. above 80 years of age, or discharged to a health care institution) in our cohort. However, resources would not permit pre-contacting all patients. Also, a closer scrutiny of the Cochrane review revealed that despite a highly significant (*p*<0.001) moderate pooled odds ratio (OR = 1.45) in favour of pre-contact, heterogeneity of study populations, reported odds ratios, and types of questionnaires in the studies included in the meta analysis was substantial.

Of the 45 papers reporting on pre-contact vs. no pre-contact on final response rates, we found very few studies targeting a population similar to our population of elderly patients. The studies were rated from A to C according to Cochrane’s standard quality assessment grading scheme ([1], p. 3). Only 5 studies were A-rated. Furthermore, the review contained information about the various studies’ target groups, and the nature of the surveys. Only 15 of the 45 papers included in [1] had a health perspective; only 8 of these targeted patients. Of the 15 health related studies, there were 6 investigating pre-contact by phone, and 9 investigating pre-contact by mail. Restricting ourselves to A- or B-rated patient-targeted pre-notification by telephone, there were one B-rated study [3] which found found a positive effect (OR = 2.58 [1.50,4.44]) of pre-contact by telephone on response rates, and one A-rated study [4] which found a positive effect (OR = 1.27 [1.00,1.63]). However, the target populations were young and mid-life women; rather dissimilar to our senescent stroke patients.

From our health services researcher’s perspective we concluded that even though the pooled data from [1] yielded a statistically significant and positive effect of pre-contact, we were unsure if it would be efficient with a fairly comprehensive questionnaire eliciting sensitive information in a population like ours. We therefore decided to undertake a randomized controlled trial (RCT), with the aim of establishing whether pre-contact by telephone increases response rates to postal questionnaires in a frail and senescent patient population.

## Methods

### RCT

The RCT was prospectively registered with Current Controlled Trials [5] on May 25th 2013 with International Standard RCT Number http://www.isrctn.com/ISRCTN31304930. The study was conducted between June 1st 2013 and April 1st 2014. The RCT was designed as an interventional open randomized controlled trial with two study branches – one *intervention group (IG)* and one *control group (CG)* – and main outcome was performed with a two-sided test for equality of proportion (significance level of *α*=0.05), analysed as per *intention-to-treat (ITT)*. Main outcome was response rate after 45 days; secondary outcome was response rate after 365 days.

### Inclusion

All patients discharged from the SU with a stroke diagnosis between May 15th 2012 and April 1st 2013, who:

1. had not previously been pre-contacted;
2. had not returned the 3-month questionnaire, or, had not received them;
3. were not pre-selected for care-giver contact, i.e. were either above 80 years of age or were discharged to other than home address with primary care-giver at other address than themselves;
4. were registered with a phone number for contact.

With regard to items 1. and 3. recall that the RCT was carried out in conjunction with the *second* round of survey questionnaires mailed to this population. We had already defined a protocol for pre-contacting the primary caregivers of the most frail patients. As these most frail patients were also to be pre-contacted prior to mailing the 12 month questionnaire, they were not eligible for inclusion into the RCT. Item 2. was included in the protocol because we expected that the effect of the intervention would be stronger amongst those patients who were known non-responders at the 3 month follow-up (This inclusion criterion has also been employed in a B-rated study by Ogborn et. al. [6], in which they reported an OR of 1.86 in favour of pre-contact in a non-responder population).

### Mailing of questionnaires and intervention

All patients in the study received an envelope with hand-written address in the name of the patient, and, where available, also the name of the spouse. The envelope contained two questionnaires and a cover letter explaining the purpose of the study, ensuring confidentiality, and equipped with contact information for the second author (TBS) in case the recipient had any queries. The cover letter was hand-signed by TBS. The two questionnaires were made out for the patient and for a care giver; such as a spouse, a child, or another close relative or friend. The questionnaires had 16 (patient) and 8 (care giver) pages, and contained questions about history of contacts with various parts of the health care system, self reported general and mental health (modified Rankin scale, EQ-5D, HADS, Barthel ADL), knowledge about stroke, satisfaction with municipal services, and help received from/provided by care-givers. The questionnaires were estimated to take 30–60 minutes to complete. The shipment also contained two stamped and addressed return envelopes; one for the patient’s questionnaire and one for the care giver’s questionnaire. Prior to mailing the questionnaire, the status of the patient was verified with the electronic patient journal system of the hospital; i.e. whether the patient was still alive, had had a change of address or were currently admitted to hospital.

The above describes the standard protocol for the NORSPOT project.

Those who were randomized into the IG were pre-contacted by TBS according to the following protocol:

- Three attempts were made at contracting the subjects at their mobile telephone number (or land-line when no mobile telephone number was available).
- If contact was established during one of these three attempts, or on a call-back, we aimed at achieving what we refer to as *an informal consent to shipping [of the questionnaire].* This means that the caller will aim not at obtaining consent to use the collected data for research (this could be submitted on the back of the questionnaire), nor to persuade or obtain a promise of return, but only to have the participant agreeing to having the questionnaire shipped for their review.
- The caller answered questions about the survey, but remained neutral towards anything but the aim: obtain the consent to shipment.
- Any house-hold member [in particular when a land-line was used] could act as proxy for the participant, e.g. a spouse or other family member answering the phone.
- Whenever a participant/proxy was unwilling to grant the *informal consent to shipping* the participant would not have a questionnaire mailed to their address. Such participants were logged with REFUSED.
- Where a prior written consent to participation (given at the SU) existed, the caller could remind the participant of this fact. However, the caller were required to take all possible measures not to induce guilt for not having responded to the 3-month shipment.

Note that the above protocol for pre-contact was already de facto established, but then aimed primarily at care givers; see item 3. in the Inclusion-section.

### Randomization and analyses

Each participant was assigned an internal identification number. A list of the participants identification numbers were sent to a statistician external to the project (see Acknowledgements) who generated the CG and the IG by random allocation. This was performed in two rounds: first in June 2013, and consecutively a second batch of non-responders from the three-month round were included and randomized to the CG and IG in September 2013.

The main analysis of the effect of the pre-contact intervention on response rates is performed according to the principle of *intention to treat* for both the primary and the outcomes. This means that the response rates calculated for the IG include those who could not be reached by telephone and patients who refused to have a questionnaire sent to them when contacted. This means that also subjects whom we tried, but were not able to, contact are recorded as in the intervention group in the main analysis. However, RCT-participants who were discovered to be either dead, terminal or diagnosed with dementia were excluded at the time of the status check which was routinely performed for *all* NORSPOT participants.

The primary outcome is defined as response within 45 days from the day when the questionnaire was mailed; the secondary outcome is defined as response within 365 days. Experience from the 3 month questionnaires showed that no participants had responded later than 3 months after receiving the questionnaires. The secondary outcome would therefore very likely be the absolute response rate, and would reveal it if a speed-up effect masqueraded as a true increase in final response rates. Also, for some types of surveys, the timing of the information is paramount, and old information is worthless. Therefore both outcomes can be relevant in different situations.

Return date was defined as the date written by the respondent on the questionnaire. This definition was chosen because the company handling the return of the questionnaires delivered them in batches of 10 or more at a time. This meant that some of the questionnaires would be waiting for some days prior to arriving at the hospital. We therefore decided that the date-of-completion was the most uniform measure of return.

Odds ratios and two-sided significance tests (Fisher’s exact with mid-p correction and *χ*^2^-tests) were computed with the oddsratio-function of the epitools-package [7] within the statistical environment R [8]. We report *p*-values w.r.t. all three tests.

Basic descriptive statistics (age, sex-distribution, type of stroke and the ratio of participants living with a spouse) for the participants were computed for the sample as a whole, and for the IG and the CG separately: The latter values were also tested for significant differences with a standard two-sided t-test. The variable living with a spouse was included because the main protocol for mailing questionnaires involved printing the name of the spouse in addition to the name of the patient wherever a registered spouse existed on the same address. This practise originated in a hope that it would be more likely that the questionnaires were opened and returned when two individuals were named on the envelope.

A binary logistic model was also fitted to the combined data set, with responding as the dependent variable, and with predictors age, sex, type of stroke, a dummy for living with a spouse, and a dummy for being in the pre-contact group (i.e. by ITT).

Finally, a simple Cost Utility Analysis (CUA) reporting the cost per additional reply associated with this strategy for increasing the response rates is also provided.

### Ethics

The NOR-SPOT project has been submitted to a regional ethics committee for medical research – Regional Committee for Medical & Health Research Ethics South East Norway, Section B – and exempted from evaluation because the study hypotheses were not directly related to health and illness. Under Norwegian law the project should then be approved by the hospital’s internal Privacy Ombudsman. The RCT was approved prior to initation of recruitment, with Ref.No. 11.076. Any queries should be directed to the Privacy Ombudsman at Akershus University Hospital.

The trial subjects of the prenotification RCT had *not* given written consent to partake in it. This follows from the nature of the study: trying to obtain answers. More importantly, under the original NORSPOT project, ethical approval for sending out the questionnaires was approved without the necessity of obtaining prior consent.

## Results

### Main results

**Fig. 1:**
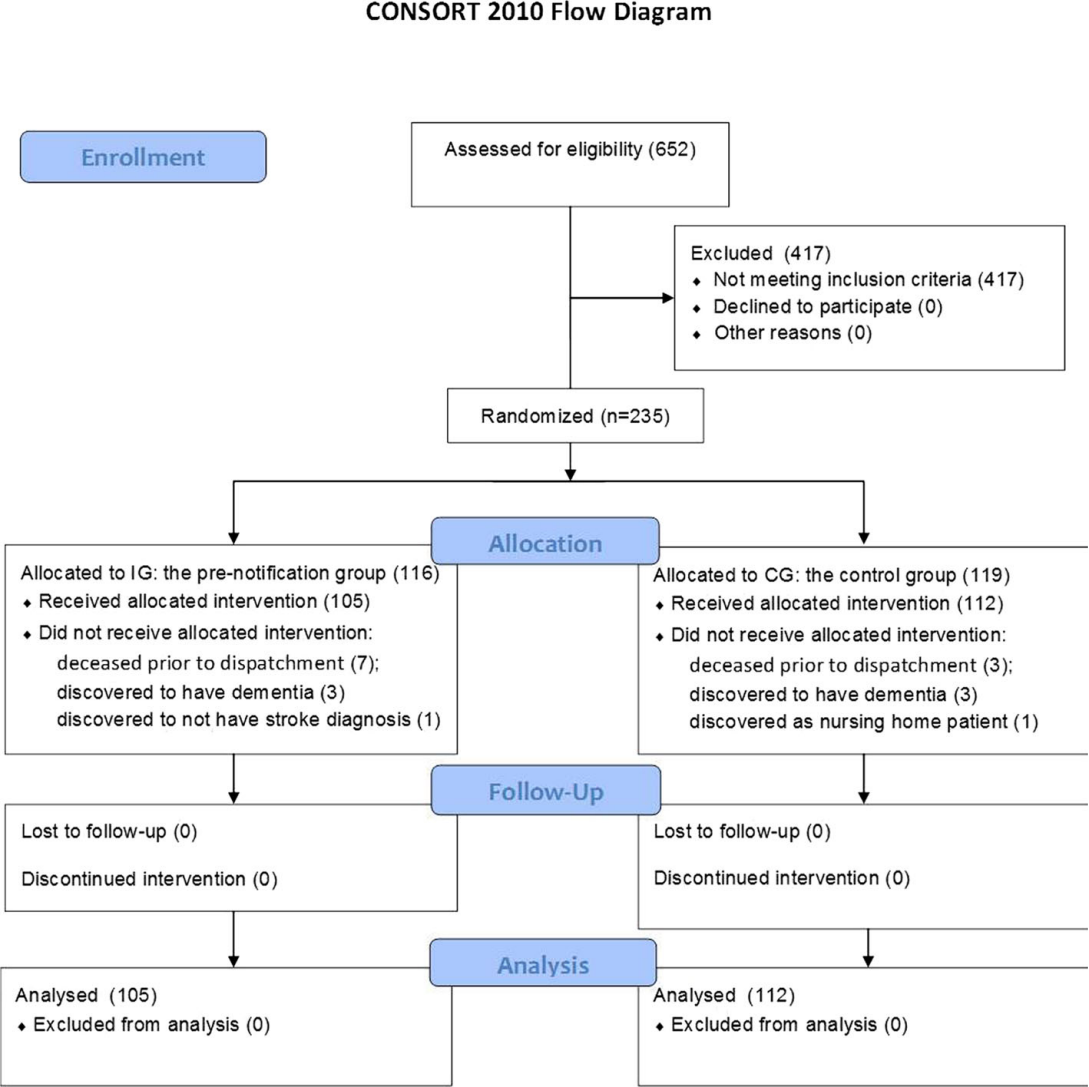
CONSORT 2010 flowchart for the inclusions and distribution of subjects to the IG and CG

A total of 235 patients, out of 652 patients assessed, were found eligible for randomization; 116 (49.4 %) were assigned to the IG and 119 (50.6 %) to the CG. During the intervention period, it was discovered through the ongoing data collection that 6 patients (3 in each arm) suffered from known dementia, and these were excluded from the RCT. 10 patients (7 in the IG and 3 in the CG) died before 12 months had passed from discharge from the SU; these were also excluded. Furthermore, one patient in the IG was discovered to have been misdiagnosed with stroke, and one patient in the control group was discovered to be a very frail nursing home patient, who had been mis-assessed as eligible for inclusion. 105 subjects in the IG and 112 controls remained (see Fig. 1). Basic descriptives of the subjects are given in Table 1; the control- and the intervention groups appear balanced with respect to age, sex, living with a spouse and type of stroke (as classified by Transient Ischemic Attack (TIA), cerebral infarction (INF), and intra-cerebral haemorrhage (ICH)).

**Table 1:** Descriptive statistics of RCT participants

	All Participants		IG		CG		
	*N*	%	*N*_IG_	%	*N*_CG_		
	217	100 %	105	48,4 %	112	51,6 %	
	Mean	sd	Mean	sd	Mean	sd	*p*
Age	63,1	13,0	64,1	13,1	62,1	13,1	0,274
	*n*	%	*n*_IG_	%	*n*_CG_	%	*p*
Male	143	65,9 %	67	63,8 %	76	67,9 %	0,532
Partner	139	64,1 %	69	65,7 %	70	62,5 %	0,624
TIA	81	37,3 %	43	41,0 %	38	33,9 %	0,288
INF	122	56,2 %	55	52,4 %	67	59,8 %	0,272
ICH	14	6,5 %	7	6,7 %	7	6,2 %	0,901

Simple descriptive statistics for the RCT participants and for the intervention- and control groups. Mean age and standard deviation of samples. The reported *p*-values are w.r.t. Welch 2-sided t-test for difference of mean/proportion between the IG and the CG computed with the native R t.test-function

The main results of the intention to treat analysis for the primary outcome and secondary outcome *first response* – defined as response returned within 45 days of the mailing date – are presented in Table 2. A clear positive effect of pre-contact by telephone was measured in both the primary and the secondary outcomes, with ORs of 2.040 (*p*=0.013) and 2.095 (*p*=0.009) respectively.

**Table 2:** Main result: ITT-analyses of response rates

	1. Outcome (45 day RR.)				2. Outcome (365 day RR.)			
	Resp	Non-Resp	Total	RR	Resp	Non-Resp	Total	RR
IG	45	60^a^	105	42.9 %	48	57	105	45.7 %
CG	30	82	112	26.8 %	32	80	112	28.6 %
Total	75	142	217^b^		80	137	217	
Test	*p*	OR [95 % CI]:		*Δ*RR	*p*	OR [95 % CI]:		*Δ*RR
Mid-p	0.014	2.040 [1.157, 3.639]		16.1 %	0.009	2.095 [1.197,3.707]		17.1 %
Fisher’s	0.015				0.011			
*χ* ^2^	0.013				0.009			

The table contains the 2x2-contingency tables w.r.t. 1. and 2. outcomes, with total figures for respondents (Resp) and non-respondents (Non-Resp) in the intervention- and control groups. Response rates (RR) for the two study arms are also provided. Below the contingency tables we report the OR with 95 % CI’s, the absolute difference in response rates (*Δ*RR), and the *p*-values from three standard 2-sided tests for effect provided by the oddsratio-function from the epitools-package for R (CI’s are computed w.r.t. the mid-*p* value)

^a^Includes the 5 respondents who were successfully contacted, but refused participation in the questionnaire study

^b^Originally 235 patients were included; 10 patients (7 from the IG and 3 from the CG) died before it was time to send them a questionnaire; 6 (3 in the IG and 3 in the CG) were discovered to be demented; 1 IG patient was discovered to have been mis-diagnosed with stroke, and one CG patient was discovered to have been mis-assessed as eligible. These 18 were excluded prior to analysis

### Secondary results

Of the 105 participants assigned to the IG we were able to successfully contact 92 (87.6 %) patients (or their care giver); 5 contacted participants (5.4 % of those reached; 4.8 % of the IG) refused to give the sought-after *informal consent to shipping* (see the Mailing of questionnaires and intervention-section). The 13 (12.4 %) participants whom we were unable to reach on the three attempts specified by the protocol were re-classified as controls (transferred to the CG) for the secondary APP-analysis. The result of this analysis is presented in Table 3, and reinforce the result from the ITT-analysis, demonstrating a positive effect of pre-contact by telephone on both the 1. and the 2. outcome, with ORs of 2.535 (*p*=0.001) and 2.354 (*p*=0.003) respectively.

**Table 3:** *As Per protocol*-analysis

	1. Outcome (45 day RR.)				2. Outcome (365 day RR.)			
	Resp	Non-Resp	Total	RR	Resp	Non-Resp	Total	RR
IG	43	49^a^	92	46.7 %	45	47	92	51.1 %
CG	32^a^	93	125	25.6 %	36	89	125	28.8 %
Total	75	142	217^b^		81	136	217	
Test	*p*	OR [95 % CI]:		*Δ*RR	*p*	OR [95 % CI]:		*Δ*RR
Mid-p	0.001	2.535 [1.432, 4.540]		21.1 %	0.003	2.354 [1.343, 4.167]		22.3 %
Fisher’s	0.001				0.003			
*χ* ^2^	0.001				0.002			

The table shows the 2x2-contingency tables w.r.t. 1. and 2. outcomes, with total figures for respondents (Resp) and non-respondents (Non-Resp) in the intervention- and control groups as defined in the APP-analysis. Response rates (RR) for the two study arms are also provided. Below the contingency tables we report the OR with 95 % CI’s, the absolute difference in response rates (*Δ*RR), and the *p*-values from three standard 2-sided tests for effect provided by the oddsratio-function from the epitools-package for R (CI’s are computed w.r.t. the mid-*p* value)

^a^The IG non-responders includes the 5 respondents who were successfully contacted, but refused participation in the questionnaire study; the CG responders includes 2 responders who were randomized to be pre-contacted, who were *not* successfully reached, but who nevertheless returned their questionnaires

^b^Originally 235 patients were included; 10 patients (7 from the IG and 3 from the CG) died before it was time to send them a questionnaire; 6 (3 in the IG and 3 in the CG) were discovered to be demented; 1 IG patient was discovered to have been mis-diagnosed with stroke, and one CG patient was discovered to have been mis-assessed as eligible. These 18 were excluded prior to analysis

The fitted binary logistic regression model yielded an OR in favour of telephone pre-contact of 2.00 (*p*=0.02) when adjusted for age, sex, type of stroke and a dummy which coded for *living with a spouse*. All the control variables – except the spouse-dummy – were insignificant. The independent OR (adjusted for pre-contact, age, sex and type of stroke) from the binary logistic regression model for responding when living with a spouse was 2.34 (*p*=0.01).

A rudimentary CUA analysis shows that the only significant resource consumed by the intervention is the time spent by the researcher/assistant. The cost of the telephone calls today are negligible (perhaps 10 cents per answered call). We therefore price the intervention in man-hours here. Due to the nature of our survey, it was important to ensure that patients had neither relocated nor were institutionalized, and, that they were still alive. Hence the book-keeping of whom we had tried to call, whom we still needed to call etcetera, and the actual calls themselves were the main driver of additional costs. Conversations could last from a few minutes to half-an-hour with patients who often took the call as an opportunity to talk and ask other questions, but we estimate that the difference in time spent on mailing questionnaires to participants in the IG compared to those in the CG was circa 10 minutes per participant. This figure was not systematically recorded, but TBS estimates that 7–8 minutes are actual time spent, whereas 10 minutes is a liberal guesstimate. An increase of 16.1 percentage points in response rate thus translates into a cost of $\frac {10\;\text {minutes}}{16.1\,\%}=62\;\text {minutes}$ per extra returned questionnaire. The monetary cost is therefore sensitive to the cost of labour qualified for performing the intervention.

## Discussion

The RCT provides evidence supporting the hypothesis that pre-contact by phone increases response rates in postal surveys targeting a patient population with a low baseline response rate. Our population was one of non-responders from the 3-month questionnaire, and the CG response rate was 27 %. The IG had a significantly increased response rate, to 43 %. We expect the result to be fairly generalizable. This is not to say that one can necessarily expect the same OR for all populations, since e.g. a population with a high baseline response rate, might be less, or more, susceptible to stimulus. The fact that our study targeted a population of non-responders – that is, subjects with a low probability of responding as reflected in the CG’s RR of 27 % – could mean that the pre-contact intervention has a higher effect in our population, than on a population of people with higher baseline propensity towards responding.

The finding that living with a spouse was revealed as a strong and independent predictor of response in the secondary analysis is also interesting. The fact that pre-contact remained a strong predictor for response after correcting for a spouse strengthens the main result.

The APP-analysis yielded a higher OR (2.535) in favour of pre-contact than the ITT-analysis (2.040). This might mean that further efforts to reach those participants in the IG whom we did not reach could strengthen the results. However, being a non-responder might be correlated with being hard to reach. We have not attempted to describe the 15 out of the 105 (14.3 %) whom we could not reach, apart from noting that their 45-day and 360-day response rates were 13 % (2) and 20 % (3) respectively, and as such not deviating much from the response rate of the CG.

We end with a few comments with regard to the CUA. Clearly the intervention’s cost depends strongly on the cost of labour; under Norwegian conditions the price is circa 40 Euro for one labour-hour. It is well-known that pecuniary incentives has a very strong and near guaranteed effect on response rates, so that one might obtain the same increase by merely paying for responses. However, adding money to the mix sometimes have unexpected results, and there is no guarantee that paying would help on a population of patients, who might consider the effort not worth the price offered. Prepaid incentives (provide incentives together with the questionnaire) are known to be efficient; see [1] for an array of further references and summarized pooled ORs. However, to beat pre-contact with this latter strategy, the offered prepaid incentive per questionnaire could not exceed the cost of 10 min of labour (under Norwegian conditions about 7 Euro), given that overall reported pooled ORs for monetary incentives are in the same range as the pre-contact OR reported here. The meta-analysis [1] also documents that larger monetary incentives yield better RR than smaller incentives, which might give pre-contact a further advantage due to its relatively low cost.

Regardless of possible local ethical considerations, it would surely be interesting to compare our reported cost-effectiveness with that of pecuniary incentives. Also, response biases could differ between the two approaches, but these questions were outside of the scope of our present study.

The bottom line is perhaps an ethical one: adding money to the equation could be conceived as inappropriate when eliciting sensitive information from a patient population, while contacting them directly to obtain their permission – appealing to a sense of companionship rather than a mercantile one – seems more in the spirit of health services research.

## Conclusion

The results are in line with previous research, and show that pre-contact has a positive effect on response rate: the estimated OR in our population was 2.04 (95 % CI [1.16,3.64]). Given the importance of a high response rates in surveys, the stipulated cost of 1 working hour per additional response is likely to be worth while; in particular if pecuniary incentives are off the table.

Protocol available from http://www.isrctn.com/ISRCTN31304930.

## Abbreviations

ABB: Abbreviation
APP: As-per-protocol
CG: Control group
CUA: Cost-utility analysis
GP: General practitioner
ICH: Intra cerebral haemorrhage
INF: Cerebral Infarction
IG: Intervention group
ITT: Intention-to-treat
OR: Odds ratio
RCT: Randomized controlled trial
RR: Response rate
SU: Stroke unit
TIA: Transient Ischemic attack
